# Patient-reported outcomes of quality of life before and after autologous hematopoietic stem cell transplantation for multiple sclerosis

**DOI:** 10.1177/13524585251315363

**Published:** 2025-02-17

**Authors:** Ann-Christine Mitrache Desaga, Yassine Noui, Thomas Silfverberg, Kristina Carlson, Joachim Burman

**Affiliations:** Department of Medical Sciences, Uppsala University, Uppsala, Sweden; Department of Medical Sciences, Uppsala University, Uppsala, Sweden; Department of Medical Sciences, Uppsala University, Uppsala, Sweden; Center for Clinical Research Dalarna, Uppsala University, Falun, Sweden; Department of Medical Sciences, Uppsala University, Uppsala, Sweden; Department of Medical Sciences, Uppsala University, Uppsala, Sweden

**Keywords:** Autologous hematopoietic stem cell transplantation, MSIS-29, multiple sclerosis, patient-reported outcomes, quality of life

## Abstract

**Background:**

Autologous hematopoietic stem cell transplantation (AHSCT) is a therapeutic intervention for multiple sclerosis (MS) that has gained increased attention in the last decade. The impact of this intervention on the quality of life (QoL) of patients with MS remains unclear.

**Objective:**

The aim of this study was to investigate the impact of AHSCT on QoL in patients with MS using Multiple Sclerosis Impact Scale (MSIS-29) scores.

**Methods:**

In this observational retrospective cohort study, patients with relapsing-remitting MS treated with AHSCT in Sweden from 2004, when the first transplant was performed, until 31 December 2019, were considered for participation. Anonymized outcome data were extracted from the Swedish MS registry in May 2022.

**Results:**

Out of 213 patients assessed for eligibility in the study, 96 were included in the final analysis. After a median follow-up of 5.2 (IQR 3.2–6.8) years, 58% improved, 14% remained unchanged and 28% worsened in the physical domain of the MSIS-29. In the psychological domain, 63% improved, 18% remained unchanged and 19% worsened. Improvements in both domains occurred early, within the first year following intervention.

**Conclusions:**

Treatment intervention with AHSCT is associated with a clinically meaningful improvement in QoL.

## Introduction

There is growing evidence that autologous hematopoietic stem cell transplantation (AHSCT) is a safe and effective treatment alternative for patients with active relapsing-remitting multiple sclerosis (RRMS) and it continues to be a promising alternative to traditional disease-modifying therapies (DMTs). The goal of AHSCT is to achieve an immunological ‘reset’ by ablation of the immune system. Current evidence supports the use of AHSCT in RRMS while active inflammatory processes are still ongoing.^1,2^ The patients most likely to tolerate and benefit from AHSCT are young, ambulatory and have inflammatory multiple sclerosis (MS) activity.^3,4^ In a randomized clinical trial on patients with RRMS, a prolonged time to disease progression was observed in patients treated with AHSCT in comparison to DMTs.^5^ An important target of therapies in RRMS is disability improvement and the maintenance of ‘no evidence of disease activity’ (NEDA), which describes the absence of relapses, absence of new MRI lesions and no worsening in disability.^6^ The proportion of patients maintaining NEDA status at 5 years following AHSCT has been reported to be 73%–79%.^7^ The intervention has well-documented effect on disability, with 54%–56% of patients with RRMS showing improvement.^5,8,9^

MS is related to a multitude of symptoms that may be difficult to assess by a physician. Patient-reported outcome measures (PROMs) can be useful in capturing their impact on patients’ lives and have seen increased use in the past decade. Several instruments have been used to assess self-reported quality of life (QoL). The Multiple Sclerosis Impact Scale (MSIS-29) is the first validated measure of disease impact specific to MS. The questionnaire consists of 29 questions of which 20 address the physical impact component and 9 the psychological impact of MS. A combined score, ranging from 0 to 100 points, can be calculated or both aspects can be reported separately. A higher score indicates worse QoL.

The implication of AHSCT on the patient’s QoL, measured with the MSIS-29, is not fully known.^10^ The objective of this study was to investigate the MSIS-29 scores in patients with MS treated with AHSCT.

## Methods

### Patient cohort

All individuals with a diagnosis of MS and treated with AHSCT at any of the seven Swedish transplantation centres from 2004, when the first transplant was performed, and before 1 January 2020, were screened for participation in this study. A total of 213 patients were assessed and, after considering the exclusion criteria, the final study cohort was comprised of 96 patients. The following criteria served as reasons for exclusion: diagnosis of primary progressive MS or secondary progressive MS according to Lublin et al.^11^ at the time of transplantation, lack of patient consent for reporting of data to the European Society for Bone and Marrow Transplantation (EBMT) registry or not fulfilling the requirements of the minimal dataset. The minimal dataset entailed data on disease course of MS at the time of transplantation and treatment date and country of transplantation; and outcomes and dates of MSIS-29 and EDSS scoring, with a minimum of one measurement pre-and post-transplantation with each instrument.

### Autologous hematopoietic stem cell transplantation

The intervention consisted of AHSCT using either BEAM-antithymocyte globulin (ATG) or Cy-ATG conditioning regimens. BEAM-ATG comprised carmustine 300 mg/m^2^, etoposide 800 mg/m^2^, cytarabine arabinoside 800 mg/m^2^, melphalan 140 mg/m^2^ and ATG from rabbit (rATG) 10 mg/kg. The Cy-ATG protocol was administered for 5 days and included cyclophosphamide 200 mg/kg and rATG 6 mg/kg. For details, see Silfverberg et al.^5^

### Statistical methods

Statistical analysis of data was performed using GraphPad Prism (v.10.2.0). Numerical values were tested for normality using D’Agostino’s test. Data were summarized using frequencies for categorical variables, means (± standard deviation, SD) for normally distributed variables and medians (interquartile range, IQR) for variables that were not normally distributed. Correlations were described with Spearman’s r and classified according to the British Medical Journal guidelines.^12^ A multivariate regression model was constructed to determine whether age, sex, disease duration, date of transplant, EDSS scores and previous treatments influenced the MSIS-29 scores. The Chi-square test was used to establish statistical significance when comparing categorical variables. Student’s t-test was used to compare normally distributed data, and Mann–Whitney test for non-parametric data. The Wilcoxon signed-rank test was used to establish statistical significance (*p* value < 0.05) between two time points.

Based on current literature, the threshold for a clinically meaningful change was set as a change in 8 points in the physical domain and 6 points in the psychological domain.^13,14^

Assessments were analysed at baseline and at years 1, 2 and 3 (±180 days) following the intervention, as well as at the latest evaluation.^15^

## Results

### Study cohort

**Figure 1.**
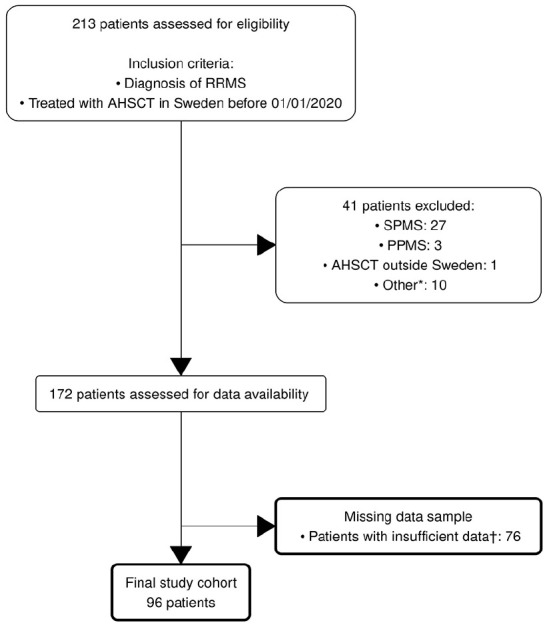
Flowchart diagram of patient inclusion and exclusion in the study. *AHSCT performed for lymphoma (*n* = 1), multiple myeloma (*n* = 1), neuromyelitis optica (*n* = 1), chronic inflammatory demyelinating polyradiculoneuropathy (*n* = 1), co-existing stroke (*n* = 1), insufficient follow-up data (*n* = 3) and not within time frame (*n* = 2). †Did not meet the criterion of at least one MSIS-29 measurement before AHSCT and another MSIS-29 measurement after AHSCT.

Data were exported from the Swedish MS registry (SMSreg) on 22 May 2022. Of the 213 considered for the study, 41 were excluded due to diagnosis other than RRMS or due to treatment outside of Sweden. A further 76 were excluded prior to the final analysis because they did not meet the criterion of at least one MSIS-29 and one EDSS measurement before and after AHSCT (Figure 1). These 76 constitute the missing data group. The final study cohort was comprised of 96 patients, 63 females and 33 males. The median age was 31 (IQR 27–36) years, with the youngest patient being 20 years and the oldest 51 years old (Table 1). The median disease duration before performing AHSCT was 4 (IQR 2.0–6.8) years. The median number of MSIS-29 assessments was 7 (IQR 5.0–10) and the median number of follow-up years was 5.2 (IQR 3.2–6.8). A total of 1118 MSIS-29 scores were analysed.

**Table 1.** Patient characteristics and details.

Characteristics	Final study cohort, *n* = 96	Missing data sample, *n* = 76
**Sex** *n* **(%)**		
Male	33 (34)	28 (37)
Female	63 (66)	48 (63)
**Age (years)**		
Mean (standard deviation, SD)	33 (±7.2)	31 (±8.4)
**Disease duration (years)**		
Median (interquartile range, IQR)	4.0 (2.0–6.8)	2.0 (1.0–8.0)
**MSIS-29 assessments**		
Median (IQR)	7.0 (5–10)	n/a
**EDSS at baseline**		
Median (IQR)	3.25 (2.0–4.0)	3.5 (2.0–5.0)
**EDSS at 1** **year**		
Median (IQR)	2.5 (1.5–3.5)	2.0 (1.0–3.0)
**Follow-up years**		
Median (IQR)	5.2 (3.2–6.8)	6.7 (4.3–9.9)
**Date of transplant (year)**		
Median (IQR)	2016 (2014–2018)	2014 (2011–2016)
**Last therapy before transplant** *n* (%)		
Natalizumab	34 (35)	15 (20)
Rituximab	28 (29)	13 (17)
Beta-interferons	2 (2)	12 (16)
Fingolimod	12 (10)	3 (4)
Glatiramer acetate	2 (2)	6 (8)
Dimethylfumarate	3 (3)	2 (3)
Intravenous immunoglobulin	0 (0)	2 (3)
Teriflunomide	2(2)	0 (0)
Alemtuzumab	3 (3)	2 (3)
Mitoxantrone	0 (0)	4 (5)
None	5 (5)	4 (5)
**Conditioning regimen** *n* (%)		
BEAM**-**ATG	15 (16)	26 (35)
Cy-ATG	81 (84)	50 (66)

A higher degree of patients had received BEAM as a conditioning regimen in the group with the missing data. In the final study cohort, 16% had received BEAM, in contrast to 35% in the missing data group (Table 1).

### Prior treatments

The median number of MS treatments prior to AHSCT was 2 (IQR 2–4). These included natalizumab (*n* = 63), rituximab (*n* = 41), beta-interferons (*n* = 60), intravenous immunoglobulins (*n* = 8), alemtuzumab (*n* = 3), glatiramer acetate (*n* = 16), teriflunomide (*n* = 4), fingolimod (*n* = 28), mitoxantrone (*n* = 2) and dimethylfumarate (*n* = 10). Some patients had received multiple therapies prior to their transplant. The most common last therapy administered prior to AHSCT was natalizumab (*n* = 34, *n* = 15). Only four patients had not used any DMT prior to AHSCT.

### Analysis of the missing data sample

The main difference between the final study cohort and the group of patients with missing data was the time point for the intervention, which was considerably later in the final study cohort, median 2016 (IQR 2014–2018) versus 2014 (2011–2016). This was also reflected in the higher proportion of patients who had used high-efficacy DMTs in the final study cohort (natalizumab, 35% vs 20%, and rituximab, 29% vs 17%) and also in the proportion of patients receiving the Cy/ATG conditioning regimen (84% vs 65%).

A significant difference was observed in change in EDSS outcomes score at year 1. The missing data group experienced greater improvement than the final study cohort. The median change in EDSS score from baseline to year 1 in the final study cohort was −0.5 (*n* = 91, *p* < 0.0001) and −1.5 (*n* = 62, *p* < 0.0001) in the missing data group.

### The physical and the psychological domain of the MSIS-29 correlated well with each other

The physical impact score correlated well with the psychological score at baseline (*r* = 0.76, *p* < 0.0001). Similarly, there was a strong correlation between the two at each follow-up: year 1 (*r* = 0.70, *p* < 0.0001), year 2 (*r* = 0.77, *p* < 0.0001), year 3 (*r* = 0.73, *p* < 0.0001) and at the latest assessment (*r* = 0.80, *p* < 0.0001).

### The EDSS correlated more strongly with the physical component of the MSIS-29

At baseline, the EDSS correlated moderately with the physical domain of MSIS-29, but less so with the psychological domain. At year 1, the EDSS correlated strongly with the physical component of MSIS-29, but only moderately with the psychological component. At year 2, the EDSS correlated very strongly with the physical component of MSIS-29, but only moderately with the psychological component. At year 3, the EDSS correlated strongly with the physical component of MSIS-29, but only moderately with the psychological component.

### The MSIS-29 increased before intervention with AHSCT

**Figure 2.**
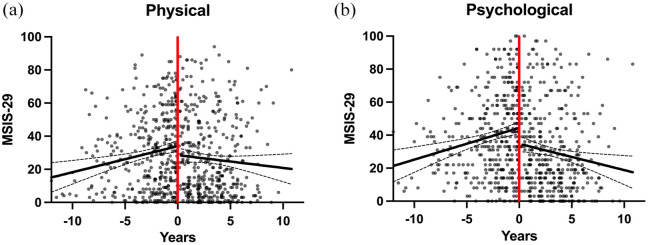
The evolution of the MSIS-29 scores over time. The evolution of the physical (a) and psychological (b) MSIS-29 scores over time with regression lines (dashed lines representing 95% confidence interval (CI)). The absolute scores are plotted on the y-axis and the number of years in relation to autologous hematopoietic stem cell transplantation is plotted on the x-axis. The MSIS-29 scores increased up until year 0 (the time of intervention) and thereafter decreased. The differences between the slopes of the regression lines were statistically significant for the physical domain (*p* = 0.0019) as well as the psychological domain (*p* > 0.0001) suggesting that intervention with autologous hematopoietic stem cell transplantation alters the evolution of self-reported quality of life.

Prior to transplant, the MSIS-29 scores increased in both domains, with a rate of 1.6 points per year in the physical and 1.9 points per year in the psychological domain (Figure 2). The mean duration until a clinically meaningful worsening occurred, following the first evaluation, was 5 years in the physical and 3 years in the psychological component. Following AHSCT, scores decreased in both domains, with a rate of 0.8 points per year in the physical and 1.6 points per year in the psychological domain.

### The MSIS-29 score decreased after AHSCT for MS

**Figure 3.**
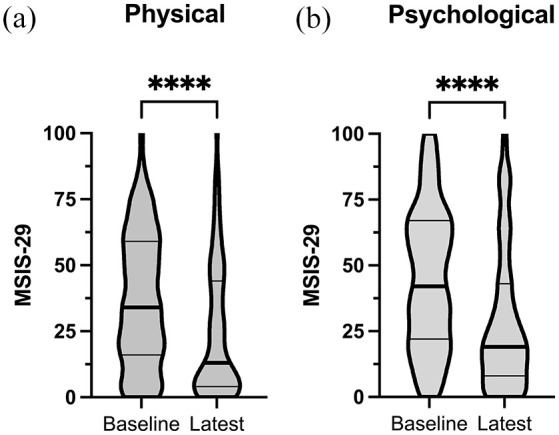
Comparison of absolute scores between baseline and latest assessment. (a) MSIS-29 physical and (b) psychological absolute scores at baseline versus the latest reported MSIS-29. After a median follow-up time of 5.2 (IQR 3.2–6.8) years, the MSIS-29 physical decreased from 34 (IQR 16–59) to 13 (IQR 4–44) and the MSIS-29 psychological decreased from 42 (IQR 22–67) to 19 (IQR 8–43) (both *p* < 0.0001).

An initial analysis of the change in physical and psychological components of the MSIS-29 over time was performed by comparing the score at baseline with the latest score (Figure 3). The median time point for the last follow-up was 4 (IQR 3–6) years from baseline. The median physical score at baseline was 34 (IQR 16–59) and at the last follow-up 13 (IQR 4–44) (*p* < 0.0001). The median psychological score at baseline was 42 (IQR 22–67) and at the last follow-up 19 (IQR 8–43) (*p* < 0.0001) (Table 2).

**Table 2.** Correlations of the EDSS with the MSIS-29.

Physical (*n*)	Spearman’s *r*	*P* value
Baseline (94)	0.45	<0.0001
Year 1 (52)	0.62	<0.0001
Year 2 (49)	0.83	<0.0001
Year 3 (40)	0.66	<0.0001
Psychological (*n*)	Spearman’s *r*	*P* value
Baseline (94)	0.21	0.047
Year 1 (51)	0.41	0.003
Year 2 (49)	0.54	<0.0001
Year 3 (40)	0.50	0.0009

A majority of patients reported a clinically meaningful improvement in the physical as well as the psychological component of the MSIS-29.

Using the set definitions of the minimum clinically meaningful change, 56 patients (58%) had improved, 13 patients (14 %) remained unchanged and 27 patients (28%) had worsened at the latest assessment in the physical component of the MSIS-29. Similarly, in the psychological component, 60 patients (63%) had improved, 17 patients (18%) remained unchanged and 18 patients (19%) had worsened.

### The MSIS-29 score decreased early after AHSCT

A clinically meaningful change in MSIS-29 score occurred early, within the first year following transplant. This holds true for both the physical and the psychological components.

The median physical score was 34 (IQR 16–59) at baseline, 15 (IQR 6–42) at year 1, 19 (IQR 4–48) at year 2 and 13 (IQR 3–33) at year 3 (Table 3). A statistically significant difference of −19 points was observed between baseline and year 1 in the physical domain (*n* = 56, *p* < 0.0001). There was no statistically significant difference between years 1 and 2 (*n* = 56, *p* = 0.9), or years 2 and 3 (*n* = 43, *p* = 0.4). There were fewer pairs when comparing years 1 and 2 (*n* = 56), as well as years 2 and 3 (*n* = 43). This is because assessments at these time points were not reported in all patients, as the available data varied from patient to patient.

**Table 3.** Absolute MSIS-29 scores.

	Baseline (*n* = 97)	Year 1 (*n* = 55*)	Year 2 (*n* = 56)	Year 3 (*n* = 43)
Physical Median (interquartile range, IQR)	34 (16–59)	15 (6–42)	19 (4–48)	13 (3–33)
Psychological Median (IQR)	42 (22–67)	25 (11–47)	26 (9–46)	19 (6–42)

Median and mean absolute score for the psychological and the physical component, and number values (*n*) year by year.

* *n* = 56 in the MSIS-29 physical domain.

The median psychological score was 42 (IQR 22–67) at baseline, 25 (IQR 11–47) at year 1 and 26 (IQR 9–46) at year 2 and 19 (IQR 6–42) at year 3. A statistically significant change of −17 points was observed between baseline and year 1 in the psychological domain (*n* = 55, *p* < 0.0001). There was no statistically significant difference between years 1 and 2 (*n* = 38, *p* = 0.4) and years 2 and 3 (*n* = 26, *p* = 0.4).

### Patients with NEDA reported higher QoL

**Figure 4.**
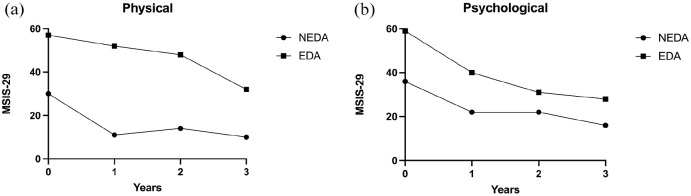
Physical/psychological absolute scores and NEDA/EDA status. (a**)** Physical and (b) psychological scores *vis-a-vis* NEDA/EDA status during follow-up at four time points: baseline, follow-up at year 1, year 2 and year 3.

At baseline, patients maintaining NEDA throughout follow-up reported a lower MSIS-29 median physical score of 30 (IQR 15–46) than patients with EDA who reported a median physical score of 57 (IQR 27–68) (*p* = 0.003) (Figure 4). Similarly, at baseline in the psychological domain, patients maintaining NEDA reported a lower MSIS-29 median score of 36 (IQR 20–63) than patients with EDA reporting a median score of 59 (IQR 29–73) (*p* = 0.045). At year 1, patients maintaining NEDA reported a median physical score of 11 (IQR 5–29) and patients with EDA had a median physical score of 52 (IQR 29–63) (*p* < 0.0001). At year 1 in the psychological domain, patients maintaining NEDA reported a median score of 22 (IQR 7–39), and patients with EDA had a median score of 40 (IQR 25–53) (*p* = 0.02). A clinically meaningful improvement in the physical domain of MSIS-29 occurred at the 1-year follow-up in the median patient maintaining NEDA and not until the 2-year follow-up in patients exhibiting EDA. In the psychological domain, a clinically meaningful improvement occurred at the 1-year follow-up, irrespective of EDA/NEDA status.

## Discussion

A key finding of this study is that a majority of patients experienced an improvement in QoL following AHSCT. The greatest improvement occurred within the first year after transplant and was maintained throughout follow-up. Patients who maintained NEDA throughout the follow-up period reported lower MSIS-29 scores than those with EDA. Patients with EDA also benefitted from AHSCT, but, on average, it took longer until a clinically meaningful improvement occurred in the physical domain. These findings contribute to our understanding of how AHSCT impacts QoL.

QoL is a useful primary endpoint for interventions, as it is a multi-dimensional construct, affected both by disease-related factors and by individual factors such as perceived self-efficacy.^16^ In recent years, the use of PROMs has increased in the field of MS and this has helped broaden the evaluation and definition of successful treatments.^17^ These tools enable the recording of health-related data from patients, with the report coming directly from the patient. One advantage of using PROMs when evaluating disability and the well-being of patients with MS is that the questionnaires can be completed from their homes and potentially decrease the need for follow-up visits. There are, however, several factors that can influence the reliability of PROMs and that require further understanding, such as the patient’s perception of well-being which may change over time and the influence of the placebo effect.^18^

The heterogeneous nature of symptoms in MS constitutes a challenge when developing PROMs. There are outcome measures that focus on specific domains and others, such as the MSIS-29, that are multi-domain measures. Unlike other instruments commonly used to evaluate QoL, such as the EQ-5D and the 36-item Short Form Health Survey (SF-36), the MSIS-29 is a disease-specific measuring tool.^19,20^ Beyond determining overall QoL, the MSIS-29 also measures disease-specific outcomes such as bladder dysfunction, participation in daily activities and fatigue. This enables the monitoring of multiple symptoms using a single instrument.^21^ In comparison to other instruments, the physical domain of the MSIS-29 has been found to have better sensitivity to discriminate between disability groups.^22^ In a British comparative study, the MSIS-29 was found to be the most responsive physical and second most responsive psychological scale.^23^ An advantage with the MSIS-29 is that there exists a generally accepted minimally clinically important difference (MCID), that can be used to estimate treatment effects.^13–15^ In contrast, no MCID has been established for the SF-36 in patients with MS, and instead, the value of half of a standard deviation is often used. This might make it less suitable to measure changes considered clinically meaningful to patients with MS.^24^ In 2022, a patient-centred standard outcome set for MS was developed, recommending the use of the MSIS-29.^25^

Several studies have examined the impact of high-efficacy DMTs on QoL in patients with MS.^7^ A decrease in MSIS-29 scores was observed in patients treated with natalizumab and rituximab, but the changes were relatively minor and not clinically meaningful for the average patient.^5,26–28^ In contrast, a clinically meaningful improvement in both domains of the MSIS-29 was seen with AHSCT in the present study. Absolute scores in both components of the MSIS-29 were decreased already at the first follow-up visit and continued to remain stable. In one previous study of 9 Italian patients with severe progressive MS, improvements in MSQOL-54 by 24 months were reported in the physical health composite as well as the mental health composite.^29^ In a mixed cohort (mainly SPMS) of 34 Brazilian patients and in a report from Lithuania of 18 patients with unknown disease course, SF-36 improved at 12 months.^30,31^ In a larger single-centre study of a mixed cohort (mainly RRMS) of 132 patients, the total SF-36 score improved from a median of 45–64, 2 years after AHSCT.^32^ These findings further support AHSCT as a viable treatment option for MS.

It is currently debated whether maintenance of NEDA is prognostic for disability in MS, and therefore we wanted to explore whether patients who maintained NEDA reported higher QoL. Somewhat unexpectedly, we found that patients with EDA during follow-up had higher MSIS-29 scores in both domains already at baseline. This probably reflects that patients with high MSIS-29 scores also have more disability and that patients with disability tend to do worse in terms of outcome after AHSCT. During follow-up, the MSIS-29 scores decreased in both domains, regardless of EDA/NEDA status, albeit slightly slower in the physical domain in patients with EDA.

This observational study is limited by the absence of a control group and therefore does not account for the potential influence of regression to the mean and the patients’ hopes and expectations.^33^ There was also a fairly large proportion of patients who did not report any MSIS-29 scores – the missing data group. This was mainly due to the limited use of the MSIS-29 before 2013. The missing data group had a larger improvement in EDSS than the patients included in the study, suggesting that our study may have underestimated the effect of AHSCT on the MSIS-29 in the entire cohort. As a result of the variable follow-up of QoL, the number of reported MSIS-29 assessments varied among patients. Finally, in Sweden, AHSCT is approved by the Swedish Board of Health and Welfare for use in aggressive RRMS, whereas SPMS and PPMS are not endorsed. Therefore, our analysis was restricted to RRMS patients and the findings cannot be generalized to people with progressive forms of MS. Some notable strengths of this study are the notably high coverage of MS cases in the SMSreg, the high data density, as well as the relatively large cohort and long follow-up.

## Conclusions

In this cohort study of patients with relapsing-remitting MS undergoing AHSCT, the treatment intervention was associated with a clinically meaningful improvement in QoL. This finding is significant as no current DMT has conclusively demonstrated a clinically meaningful long-term impact on QoL in patients with MS. Despite often being considered a last-resort treatment, offered only when other alternatives have failed, our findings support a more widespread use of AHSCT. This study underscores the potential of AHSCT as an effective treatment option for improving the QoL in patients with MS.

## References

[1] Muraro PA Pasquini M Atkins HL , et al. Long-term outcomes after autologous hematopoietic stem cell transplantation for multiple sclerosis. JAMA Neurol 2017; 74(4): 459–469. 28241268 10.1001/jamaneurol.2016.5867 PMC5744858

[2] Scolding NJ Pasquini M Reingold SC , et al. Cell-based therapeutic strategies for multiple sclerosis. Brain J Neurol 2017; 140(11): 2776–2796. 10.1093/brain/awx154 PMC5841198 29053779

[3] Thompson AJ Baranzini SE Geurts J , et al. Multiple sclerosis. The Lancet 2018; 391(10130): 1622–1636. 10.1016/S0140-6736(18)30481-1 29576504

[4] Muraro PA Martin R Mancardi GL , et al. Autologous haematopoietic stem cell transplantation for treatment of multiple sclerosis. Nat Rev Neurol 2017; 13(7): 391–405. 28621766 10.1038/nrneurol.2017.81

[5] Silfverberg T Zjukovskaja C Ljungman P , et al. Haematopoietic stem cell transplantation for treatment of relapsing-remitting multiple sclerosis in Sweden: An observational cohort study. J Neurol Neurosurg Psychiatry 2024; 95(2): 125–133. 37748927 10.1136/jnnp-2023-331864 PMC10850659

[6] Rotstein DL Healy BC Malik MT , et al. Evaluation of no evidence of disease activity in a 7-year longitudinal multiple sclerosis cohort. JAMA Neurol 2015; 72(2): 152–158. 25531931 10.1001/jamaneurol.2014.3537

[7] Sormani MP Muraro PA Saccardi R , et al. NEDA status in highly active MS can be more easily obtained with autologous hematopoietic stem cell transplantation than other drugs. Mult Scler 2017; 23(2): 201–204. 27207454 10.1177/1352458516645670

[8] Burt RK Balabanov R Burman J , et al. Effect of nonmyeloablative hematopoietic stem cell transplantation vs continued disease-modifying therapy on disease progression in patients with relapsing-remitting multiple sclerosis: A randomized clinical trial. JAMA 2019; 321(2): 165–174. 30644983 10.1001/jama.2018.18743 PMC6439765

[9] Signori A Boffa G Bovis F , et al. Prevalence of disability improvement as a potential outcome for multiple sclerosis trials. Mult Scler 2021; 27(5): 706–711. 32589486 10.1177/1352458520936236

[10] McGuigan C Hutchinson M . The multiple sclerosis impact scale (MSIS-29) is a reliable and sensitive measure. J Neurol Neurosurg Psychiatry 2004; 75(2): 266–269. 14742602 PMC1738881

[11] Lublin FD Reingold SC Cohen JA , et al. Defining the clinical course of multiple sclerosis: The 2013 revisions. Neurology 2014; 83(3): 278–286. 24871874 10.1212/WNL.0000000000000560 PMC4117366

[12] Wechsler S . Statistics at Square One. 9th ed (Rev. MJ Campbell and Swinscow TDV). London: BMJ Publications, 1996.

[13] Martínez-Lemos I Martínez-Aldao D Seijo-Martínez M , et al. Nordic walking for people with relapsing-remittent multiple sclerosis: A case series study. Mult Scler Relat Disord 2020; 46: 102479. 32911307 10.1016/j.msard.2020.102479

[14] Widener GL Allen DD . Measurement characteristics and clinical utility of the 29-item multiple sclerosis impact scale. Arch Phys Med Rehabil 2014; 95(3): 593–594. 24701634 10.1016/j.apmr.2013.07.008

[15] Costelloe L O'Rourke K Kearney H , et al. The patient knows best: Significant change in the physical component of the multiple sclerosis impact scale (MSIS-29 physical). J Neurol Neurosurg Psychiatry 2007; 78(8): 841–844. 17332049 10.1136/jnnp.2006.105759 PMC2117755

[16] Schäffler N Schönberg P Stephan J , et al. Comparison of patient-reported outcome measures in multiple sclerosis. Acta Neurol Scand 2013; 128(2): 114–121. 23398571 10.1111/ane.12083

[17] Hawton A Green C Telford C , et al. Using the multiple sclerosis impact scale to estimate health state utility values: Mapping from the MSIS-29, version 2, to the EQ-5D and the SF-6D. Value Health 2012; 15(8): 1084–1091. 23244811 10.1016/j.jval.2012.07.007

[18] van ’t Hullenaar C Coerver E Kalkers NF , et al. The use of multi-domain patient reported outcome measures for detecting clinical disease progression in multiple sclerosis. Mult Scler Relat Disord. October 2021; 55: 103165. 10.1016/j.msard.2021.103165 34404022

[19] Sharrack B Hughes RA . The guy’s neurological disability scale (GNDS): A new disability measure for multiple sclerosis. Mult Scler 1999; 5(4): 223–233. 10467380 10.1177/135245859900500406

[20] Jones KH Ford DV Jones PA , et al. How people with multiple sclerosis rate their quality of life: An EQ-5D survey via the UK MS Register. Plos One 2013; 8(6): e65640. 10.1371/journal.pone.0065640 PMC3679154 23776516

[21] Daniels K Frequin STFM van de Garde EMW , et al. Development of an international, multidisciplinary, patient-centered standard outcome set for multiple sclerosis: The S.O.S.MS project. Mult Scler Relat Disord 2023; 69: 104461. 36563595 10.1016/j.msard.2022.104461

[22] Marrie RA Dolovich C Cutter GR , et al. Comparing the MSIS-29 and the health utilities index mark III in multiple sclerosis. Front Neurol 2021; 12: 747853. 34975716 10.3389/fneur.2021.747853 PMC8718449

[23] Hobart JC Riazi A Lamping DL , et al. How responsive is the multiple sclerosis impact scale (MSIS-29)? A comparison with some other self report scales. J Neurol Neurosurg Psychiatry 2005; 76(11): 1539–1543. 16227547 10.1136/jnnp.2005.064584 PMC1739386

[24] Strijbis EM Repovic P Mostert J , et al. The MSIS-29 and SF-36 as outcomes in secondary progressive MS trials. Mult Scler 2022; 28(10): 1606–1619. 35876467 10.1177/13524585221105465 PMC9315187

[25] Hirt J Dembowska K Woelfle T , et al. Clinical trial evidence of quality-of-life effects of disease-modifying therapies for multiple sclerosis: A systematic analysis. J Neurol 2024; 271(6): 3131–3141. 38625399 10.1007/s00415-024-12366-5 PMC11136790

[26] Holmén C Piehl F Hillert J , et al. A Swedish national post-marketing surveillance study of natalizumab treatment in multiple sclerosis. Mult Scler 2011; 17(6): 708–719. 21228027 10.1177/1352458510394701

[27] Capra R Morra VB Mirabella M , et al. Natalizumab is associated with early improvement of working ability in relapsing-remitting multiple sclerosis patients: WANT observational study results. Neurol Sci 2021; 42(7): 2837–2845. 33205373 10.1007/s10072-020-04838-z

[28] Perumal J Fox RJ Balabanov R , et al. Outcomes of natalizumab treatment within 3 years of relapsing-remitting multiple sclerosis diagnosis: A prespecified 2-year interim analysis of STRIVE. BMC Neurol. 08 Juni 2019; 19(1): 116. 10.1186/s12883-019-1337-z PMC6555913 31176355

[29] Saccardi R Mancardi GL Solari A , et al. Autologous HSCT for severe progressive multiple sclerosis in a multicenter trial: Impact on disease activity and quality of life. Blood 2005; 105(6): 2601–2607. 15546956 10.1182/blood-2004-08-3205

[30] Guimarães FA Oliveira-Cardoso EA Mastropietro AP , et al. Impact of autologous hematopoetic stem cell transplantation on the quality of life of patients with multiple sclerosis. Arq Neuropsiquiatr 2010; 68(4): 522–527. 20730303 10.1590/s0004-282x2010000400009

[31] Giedraitiene N Gasciauskaite G Kaubrys G . Impact of autologous HSCT on the quality of life and fatigue in patients with relapsing multiple sclerosis. Sci Rep 2022; 12(1): 15404. 36100664 10.1038/s41598-022-19748-7 PMC9470541

[32] Burt RK Balabanov R Han X , et al. Association of nonmyeloablative hematopoietic stem cell transplantation with neurological disability in patients with relapsing-remitting multiple sclerosis. JAMA 2015; 313: 275–284. 25602998 10.1001/jama.2014.17986

[33] Barnett AG van der Pols JC Dobson AJ . Regression to the mean: What it is and how to deal with it. Int J Epidemiol 2005; 34(1): 215–220. 15333621 10.1093/ije/dyh299

